# Inter- vs intra-individual variation and temporal repeatability of escape responses in the coral reef fish *Amblyglyphidodon curacao*

**DOI:** 10.1242/bio.013508

**Published:** 2015-10-09

**Authors:** Maïwenn Jornod, Dominique G. Roche

**Affiliations:** Éco-Éthologie, Institut de Biologie, Université de Neuchâtel, Neuchâtel, CH 2000, Switzerland

**Keywords:** C-start, Damselfish, Fast-start, Habituation, Kinematics, Locomotion, Mauthner cells, Swimming

## Abstract

Fast-start escape responses are critical behaviours used by fishes during predator-prey encounters and some interactions with hetero- and conspecifics. In experimental studies, escape responses are often measured once per individual and considered representative of maximum performance. However, few studies have compared variability and repeatability in escape performances within and among individuals. Using the tropical damselfish *Amblyglyphidodon curacao*, we quantified inter- and intra-individual variation in behavioural and kinematic components of escape performance during repeated presentations of a stimulus at 15 min intervals. Individual maximum escape performance was repeatable through time, but there was considerable variation in the magnitude of responses both among and within fish. We found no evidence of habituation or fatigue due to repeated stimulations, suggesting that fish can be stimulated multiple times to ensure that an accurate estimate of maximum escape performance is obtained.

## Abstract (precis)

**Summary:** Quantification of the inter- and intra-individual variation in behavioural and kinematic components of escape performance of the tropical damselfish *Amblyglyphidodon curacao* found no evidence of habituation or fatigue due to repeated stimulations.

## INTRODUCTION

Survival following an encounter with a predator depends heavily on the prey's escape performance. To escape attacking predators, many fish species perform sudden accelerations called fast-start escape responses (Domenici, 2011). Fast-starts are extremely rapid burst swimming behaviours that are anaerobically powered and typically mediated by the Mauthner cells, a pair of giant neurons allowing very rapid (5–10 ms latency), reflex-like responses (Domenici, 2011; Eaton et al., 2001). These behaviours are also used by fishes in a multitude of contexts other than escaping from predators, such as minimizing predation risk (e.g. Domenici et al., 2014). The C-start is the main fast-start escape response used by fishes and is characterized by a unilateral contraction of the musculature, which bends the body into a distinctive ‘C’ shape (stage 1). The first stage of the C-start is often (but not always) followed by a contralateral muscle contraction during stage 2, leading to a return flip of the tail. This sequence is known as a double-bend C-start as opposed to a single-bend C-start, which excludes the return tail flip (Domenici, 2010a, 2011).

Unlike the first two stages of the fast-start escape response that generate most of the acceleration, stage 3 can be highly variable and involves continuous swimming, coasting or breaking (Domenici, 2011). Therefore, researchers typically focus on stages 1 and 2 when measuring escape performance for practical reasons. Even when excluding stage 3, the escape response is characterized by a rich set of behavioural (responsiveness, escape latency and directionality) and kinematic (turning radius, turning rate, turning angle and distance-related performance such as escape distance, speed and acceleration) components (Domenici, 2010b, 2011).

The fast-start escape response of fishes has long been considered a stereotyped behaviour, most likely because of its very short duration and critical role in predator evasion. Stereotyped behaviours are defined as exhibiting little variation across trials under a given set of conditions (Wainwright et al., 2008). However, recent studies have observed noticeable variation in the first two stages of escape responses (Domenici, 2011; Marras et al., 2011; Tytell and Lauder, 2002; Wainwright et al., 2008), suggesting that the fast-start escape response is more variable than previously thought. Importantly, several studies have shown variation among individuals (e.g. Chappell and Odell, 2004; Marras et al., 2011; Reidy et al., 2000; Tytell and Lauder, 2002) but few have examined differences in performance within individuals, particularly when fishes are stimulated more than twice (see Eaton et al., 1977; Fuiman and Cowan, 2003; Gibson and Johnston, 1995; Langerhans et al., 2004; Marras et al., 2011; Oufiero and Garland, 2009). Documenting inter- and intra- individual variation in escape performance is key to improve methodologies for accurately measuring this trait, particularly since intra-individual variability can result in biased performance estimates (Adolph and Pickering, 2008).

**Fig. 1.**
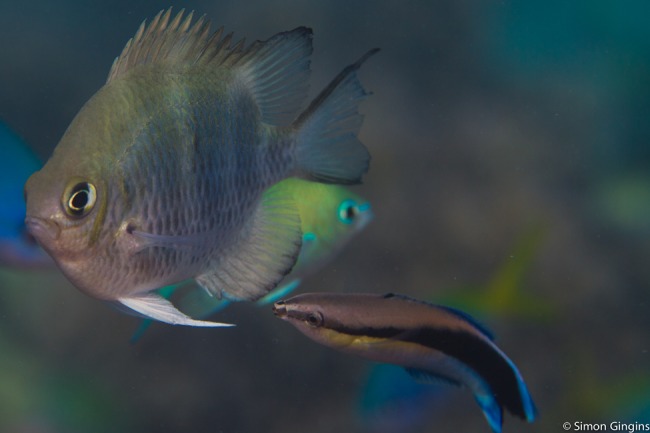
**The staghorn damsel (*Amblyglyphidodon curacao*; left) commonly uses fast-starts to escape from cheating cleaner fish (*Labroides dimidiatus;* bottom).** Instead of removing ectoparasites, cleaner fish occasionally bite their clients to feed on mucus and scales. Photo credit: Simon Gingins.

Here, we quantified and compared inter- and intra-individual variation in behavioural and kinematic components of the fast-start escape response in *Amblyglyphidodon curacao*, the staghorn damselfish (Teleostei: Pomacentridae). Other than for escaping predators, *A. curacao* commonly employs fast-starts to terminate interactions with cheating cleaner fish, *Labroides dimidiatus* (Fig. 1). Using a mechano-acoustic stimulus, we repeatedly stimulated 14 adult individuals at 15 min intervals to obtain five successive escape responses per fish. We also compared changes in the strength of escape response components across trials to test for fatigue and/or habituation to the stimulus.

## RESULTS AND DISCUSSION

**Fig. 2.**
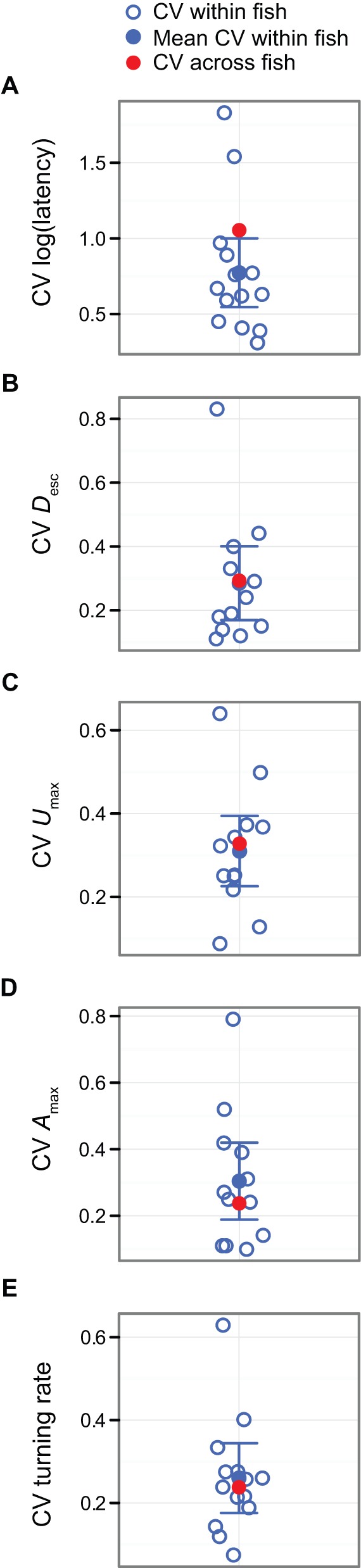
**Inter- and intra-individual variation in measures of escape performance.** Coefficient of variation (CV) for measures of escape performance for each *A. curacao* across five stimulus presentations (empty blue circles): (A) response latency (*n*=14) and (B) escape distance (*D*_esc_), (C) maximum velocity (*U*_max_), (D) maximum acceleration (*A*_max_) and (E) turning rate (*n*=12). Filled blue circles represent the mean CV within individuals; error bars are 95% confidence intervals. Filled red circles represent the CV among individuals.

Escape performance differed considerably among individual *A. curacao*, with a coefficient of variation (CV; standard deviation divided by the mean) above 0.20 for all escape response components (Fig. 2). This value is consistent with previous studies (Chappell and Odell, 2004; Marras et al., 2011; Reidy et al., 2000), and supports the idea that escape responses are not stereotyped. Behaviours are occasionally considered stereotyped when the CV among individuals is below 0.20 (and in some cases, below 0.10; Domenici, 2010a) although there is no generally accepted threshold CV below which a behaviour is defined as stereotyped (Wainwright et al., 2008). Interestingly, response latency was substantially more variable (CV=1.05) than other escape response components (Fig. 2), and higher than observed in other species (e.g. 0.375 in European sea bass; Marras et al., 2011). Some *A. curacao* had consistently high latencies (>200 ms), indicative of non-Mauthner-cell escapes (Domenici, 2011), which would explain the high variation observed (supplementary material Fig. S1). Response latency was also the only escape component measured that varied significantly more among than within individuals: the CV among individuals fell outside the 95% CI of the mean CV within individuals (Fig. 2).

**Fig. 3.**
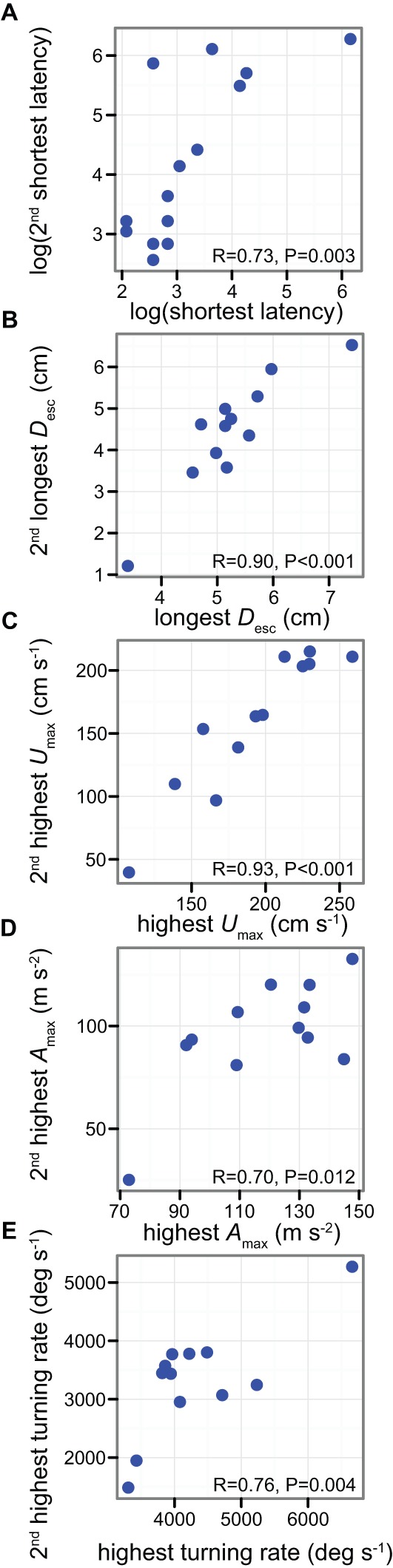
**Relationships between the highest and second highest escape performance measures in *A. curacao* across five stimulus presentations at 15 min intervals.** (A) response latency (*n*=14) and (B) escape distance (*D*_esc_), (C) maximum velocity (*U*_max_), (D) maximum acceleration (*A*_max_) and (E) turning rate (*n*=12).

Individual fish's best and second best performance for response latency, *D*_esc_, *U*_max_, *A*_max_, and turning rate were highly correlated (all R≥0.70, all *P*≤0.01) (Fig. 3). This result is consistent with previous experiments (Marras et al., 2011) and suggests that an individual's maximum escape performance is repeatable even across relatively short periods of time (Claireaux et al., 2007; Fuiman and Cowan, 2003; Marras et al., 2011; Nelson and Claireaux, 2005; Nelson et al., 2008). Importantly, however, we found considerable variation in all components of the escape response within individuals when all five stimulus presentations were considered (Fig. 2, supplementary material Fig. S1). With the exception of response latency, which varied considerably among individuals (see previous paragraph), the variation in escape performance observed among trials in the same fish (within individuals) was similar to that observed among different individuals: the 95% CI of the mean CV within individuals overlapped the CV among individuals (Fig. 2). Given the large amount of variation in the escape performance displayed by individuals between trials, our results suggest that performing multiple rather than a single stimulation will help improve the accuracy of maximum escape performance estimates (see for e.g. Langerhans, 2009; Langerhans et al., 2005, 2004; Nelson et al., 2008; Wilson et al., 2007). In lizards, for example, intra-individual variation causes an underestimation of maximum speed performance which is inversely related to the number of trials performed per individual (Adolph and Pickering, 2008). Increasing the number of trials per individual reduces this bias (Adolph and Pickering, 2008).

**Fig. 4.**
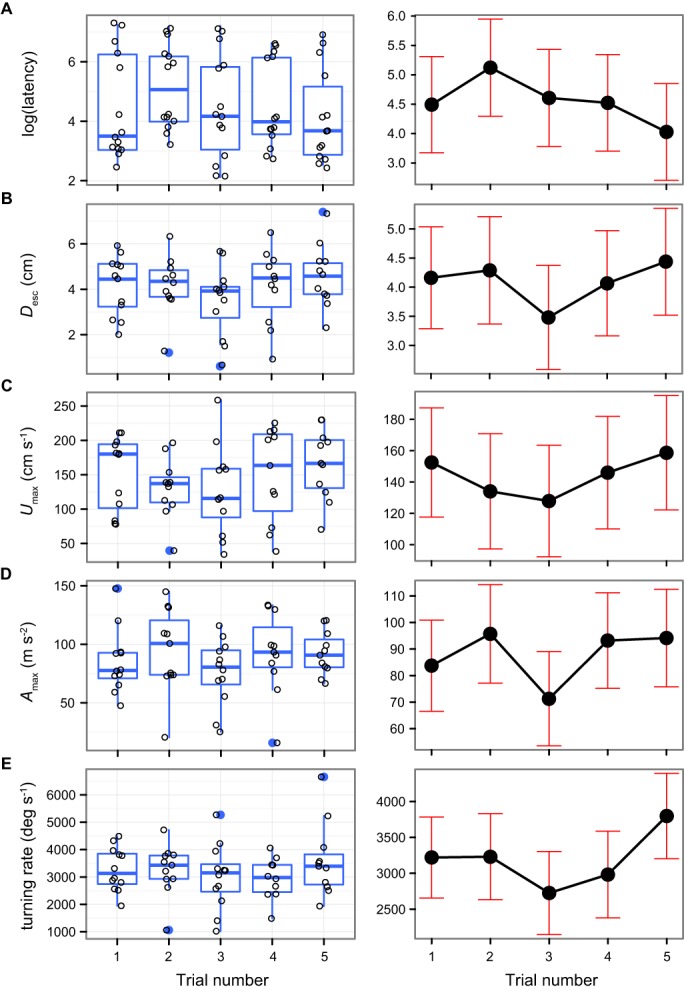
**Effect of repeated stimulations on measures of escape performance.** (A) Response latency (log transformed), (B) escape distance (*D*_esc_), (C) maximum velocity (*U*_max_), (D) maximum acceleration (*A*_max_) and (E) turning rate by trial number (*n*=5) for *A. curacao* stimulated every 15 min. The left panels (box-and-whisker plots) show the raw data; whiskers extend to the highest value within 1.5 times the inter-quartile range; data beyond the end of the whiskers are outliers and indicated as blue points. The right panels show the mean and 95% confidence intervals for each trial computed using a linear mixed-effects model (LMM; accounts for repeated measures on the same individuals through time) and visualized with the R package effects (Fox et al., 2014).

Repeating the same trial on an animal can also result in biased performance estimates if multiple stimulations lead to habituation and/or fatigue and thus a decrease in the intensity of the response. Despite repeated stimulus presentations at 15 min intervals, we found no evidence for a decline in the strength of any of the five escape response components measured. There was no effect of trial order on response latency, *D*_esc_, *U*_max_, and *A*_max_, (all *P*>0.09; Table 1, Fig. 4A-D). Turning rate increased slightly in the last trial (*F*_4,38_=3.08, *P*=0.027; Table 1, Fig. 4E). The fixed factor (trial number) and covariates (TL, distance and angle relative to the stimulus) included in the models explained only a small proportion of the variance in escape performance (between 5 and 23%); most of the variance was due to inter-individual variation among fish (i.e. the random factor fish ID; Table 1, supplementary material Table S1). Although a very short time interval (5 s) between stimulus presentations causes a reduction in the magnitude of escape responses (Eaton et al., 1977), longer time intervals between 5 and 30 min appear to eliminate any effect of habituation/fatigue (Izvekov et al., 2014; Marras et al., 2011; Tytell and Lauder, 2002). Several factors might explain why maximum performance is repeatable within a time frame of minutes. First, the neurons responsible for this behaviour have a very short refractory period of 50 ms during which activation is inhibited (Guthrie, 1987; Russell, 1976). Second, recovery from fast-start events which often last less than 100 ms and require only a few tail beats is likely to be rapid (Marras et al., 2011). For instance, the damselfish *Pomacentrus amboinensis*, which co-occurs with *A. curacao*, is capable of recovering from much longer lasting exhaustive exercise within 10 to 12 min (mean exhaustion time±s.d.=80.88±3.32 s) (Killen et al., 2014).

**Table 1.**
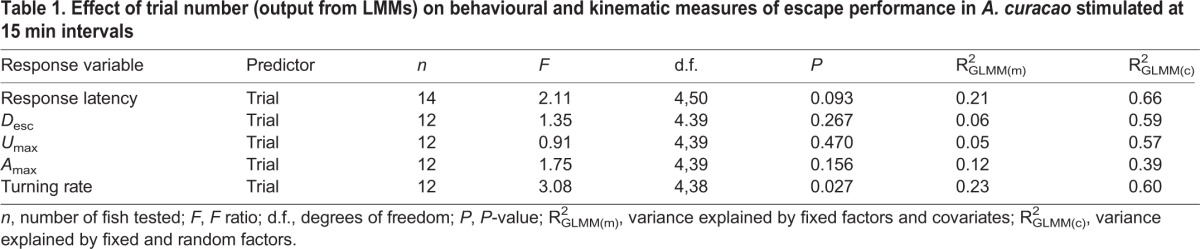
**Effect of trial number (output from LMMs) on behavioural and kinematic measures of escape performance in *A. curacao* stimulated at 15 min intervals**

In summary, we show that significant variability exists in both behavioural and kinematic components of the fast-start escape response in the tropical damselfish *A. curacao*. The magnitude of this variability was similar within and among individuals. Although performance measures are repeatable, we recommend performing several stimulations per individual when possible to improve the accuracy of maximum escape performance estimates. The number of repetitions should be positively related to the magnitude of variation within individuals (see also Adolph and Pickering, 2008). Our experiments also demonstrate that a 15 min time interval between stimulus presentations is sufficient to prevent habituation or fatigue in this species.

## MATERIALS AND METHODS

### Animals

Experiments were performed on 14 *A. curacao* (TL=11.53±0.53 cm, mean±s.d.) at the Lizard Island Research Station (14°40′S, 145°28′E) in Queensland, Australia, during September 2014. Fish were caught on reefs surrounding Lizard Island using barrier nets and housed in flow-through aquaria with water transported directly from the reef. Fish were given a 10 cm diameter PVC pipe as shelter and were fed daily with commercial fish flakes up to 24 h prior to the experiments. Research was conducted with approval from the Great Barrier Reef Marine Park Authority (G37047.1) and the Queensland Animal Ethics Committee (CA 2014/06/780).

### Experimental set-up

Fast-start trials were conducted in two large grey circular tanks (diameter=1.10 m, height=0.40 m) with flow-through seawater (6 litres min^−1^) to maintain a stable temperature (24.1±0.8°C, mean±s.d.) and high oxygen levels (>90% saturation). Each experimental tank was illuminated with two 500 W work lights placed at opposite ends of the tank. The water depth was maintained at 20 cm to minimize fish displacement in the vertical plane but allow full extension of the anal and dorsal fins. A mechano-acoustic stimulus was used to initiate escape responses. Two plastic cylinders with a tapered tip (height 12.0 cm, diameter 3.0 cm, weight 165 g) were suspended 70 cm above the water surface on either side of the tank, 13 cm from the edge (see setup in Turesson et al., 2009). The stimulus was released by switching off an electromagnet. To avoid visual stimulation before contact with the water, the stimulus fell inside an opaque PVC cylinder (outer diameter=10.3 cm) suspended 1 cm above the water surface. A high speed camera (GoPro Hero 3+ black; GoPro, San Mateo USA) was placed above the centre of the experimental tank to film escape responses at 240 Hz. The stock lens of the camera was replaced with a commercially available lens to avoid image distortion (4.14 mm f/3.0 86° HFOV 5MP GP41430; Peau Productions Inc., San Diego, USA) and could be controlled remotely with an iPad^®^ (Apple Inc., California, USA). The experimenter was shielded behind a black tarp where the camera could be operated and the electromagnet switched off. A mirror was placed under each plastic pipe to record the exact timing of when the stimulus hit the water surface.

### Experimental protocol

Each fish's centre of mass (CoM) was marked dorsally with a piece of reflective tape placed on each side of the dorsal fin (Lefrançois et al., 2005). A single fish was then placed in the experimental tank and left undisturbed for a minimum of 30 min. Fish tended to swim around the tank freely after this habituation time. To avoid variation in performance due to differences in positioning relative to the stimulus, we stimulated fish only when they were at an angle of ∼90° relative to the stimulus (81.3°±25.4°, mean±s.d.) and ∼20 cm from the edge of the PVC pipe (22.8±3.7 cm, mean±s.d.). On occasion, we used a bubble curtain that could be turned on and off to gently move the fish away from the edges of the tank. Fish were stimulated 5 times at an interval of ∼15 min (17.9±3.8 min, mean±s.d.).

### Escape performance measurements

We defined stage 1 of the fish escape response as starting with the first head movement after the stimulus and ending with the first reversal of the angular motion of the head. Stage 2 starts with the end of stage 1 and ends with straightening of the fish's body (Domenici and Batty, 1997; see line drawings in Domenici and Blake, 1997, Domenici, 2011). We analysed the videos frame by frame in ImageJ by manually tracking the CoM using the plugin MTrackJ and extracted the following: (1) response latency (the time between the first contact of the stimulus with the water surface and the first head movement of the fish); (2) distance-time variables, measured within a fixed time period (42 ms), which corresponded to the mean duration of stages 1 and 2 across all trials (see Marras et al., 2011) - these measures included escape distance (*D*_esc_) (distance covered by the CoM in 42 ms), maximum velocity (*U*_max_), and maximum acceleration (*A*_max_); (3) stage 1 turning rate, calculated as stage 1 turning angle divided by stage 1 duration (with stage 1 turning angle defined as the angle between the linear body segment running between the CoM and the tip of the snout at the beginning of the response and the same body segment at the end of stage 1). We used a five-point quadratic polynomial regression (Lanczos, 1956) to obtain smoothed values of *U*_max_ and *A*_max_ (Lefrançois et al., 2005; Marras et al., 2011).

We also measured the initial orientation of the fish relative to the stimulus (the angle between the linear segments joining the fish's CoM to the edge of the PVC pipe and the CoM to the fish's snout) and initial distance from the stimulus (distance between the CoM and the stimulus) to check for an influence of these variables on escape performance.

### Statistical analysis

We used general linear mixed-effects models (LMM) to test the effect of trial order (accumulated fatigue and/or habituation to repeated presentations of the stimulus) on five measures of escape performance: (1) escape latency (*n*=14), (2) *D*_esc_ (*n*=12), (3) *U*_max_ (*n*=12), (4) *A*_max_ (*n*=12), and (5) turning rate (*n*=12). Two fish that had consistently weak responses and long latencies were excluded from the kinematic analyses. Fish size (TL), distance to the stimulus and body angle relative to the stimulus were included as covariates in the models. Fish ID was specified as a random factor to account for the non-independence of measurements on the same individual through time. Homoscedasticity and normality of residuals were checked with plots of residuals versus fitted values and qqplots of residuals. Response latency was log transformed to meet model assumptions. We calculated the marginal R^2^ [variance explained by the fixed factors and covariates; R^2^_GLMM(m)_] and conditional R^2^ [variance explained by the fixed and random factors; R^2^_GLMM(c)_] following Nakagawa and Schielzeth (2013).

We tested repeatability following Marras et al. (2011): the best and the second best values of (1) response latency, (2) *D*_esc_, (3) *U*_max_, (4) *A*_max_, and (5) turning rate were chosen from the five escape responses for each fish, and these two values were compared for each individual using a Pearson correlation. Intraclass correlation coefficients are presented in supplementary material Table S2. We also calculated the coefficient of variation (CV; standard deviation divided by the mean) of these five performance measures for each fish, across all five stimulus presentations. The CV is a unitless measure of dispersion relative to the mean, which allows comparing the variability of different variables (Lovie, 2005). We then computed the mean CV for all fish and its 95% confidence interval, which represents the average variation *within* individuals. We compared this value to the CV across fish (computed using means for each fish), which represents variation *among* individuals, taking into account all five stimulus presentations.

Analyses were done in R 3.1.2 (R Development Core Team, 2014). Data are deposited in the public repository figshare (http://dx.doi.org/10.6084/m9.figshare.1421988).
